# Step-by-step guideline for disease-specific costing studies in low- and middle-income countries: a mixed methodology

**DOI:** 10.3402/gha.v7.23573

**Published:** 2014-03-28

**Authors:** Marleen E. Hendriks, Piyali Kundu, Alexander C. Boers, Oladimeji A. Bolarinwa, Mark J. te Pas, Tanimola M. Akande, Kayode Agbede, Gabriella B. Gomez, William K. Redekop, Constance Schultsz, Siok Swan Tan

**Affiliations:** 1 Department of Global Health, Academic Medical Center, Amsterdam Institute for Global Health and Development, University of Amsterdam, Amsterdam, The Netherlands; 2 Institute for Medical Technology Assessment, Erasmus University Rotterdam, Rotterdam, The Netherlands; 3 Department of Epidemiology and Community Health, University of Ilorin Teaching Hospital, Ilorin, Nigeria; 4 Ogo Oluwa Hospital, Bacita, Nigeria

**Keywords:** micro-costing, cost of illness, healthcare costs, methodology, developing countries, sub-Saharan Africa, global health, economic evaluation

## Abstract

**Background:**

Disease-specific costing studies can be used as input into cost-effectiveness analyses and provide important information for efficient resource allocation. However, limited data availability and limited expertise constrain such studies in low- and middle-income countries (LMICs).

**Objective:**

To describe a step-by-step guideline for conducting disease-specific costing studies in LMICs where data availability is limited and to illustrate how the guideline was applied in a costing study of cardiovascular disease prevention care in rural Nigeria.

**Design:**

The step-by-step guideline provides practical recommendations on methods and data requirements for six sequential steps: 1) definition of the study perspective, 2) characterization of the unit of analysis, 3) identification of cost items, 4) measurement of cost items, 5) valuation of cost items, and 6) uncertainty analyses.

**Results:**

We discuss the necessary tradeoffs between the accuracy of estimates and data availability constraints at each step and illustrate how a mixed methodology of accurate bottom-up micro-costing and more feasible approaches can be used to make optimal use of all available data. An illustrative example from Nigeria is provided.

**Conclusions:**

An innovative, user-friendly guideline for disease-specific costing in LMICs is presented, using a mixed methodology to account for limited data availability. The illustrative example showed that the step-by-step guideline can be used by healthcare professionals in LMICs to conduct feasible and accurate disease-specific cost analyses.

Insight into the costs of healthcare services is essential for efficient resource allocation and healthcare financing (1–5). In many cases this is informed through costing studies. An economic evaluation comprises an analysis of costs (costing study) and an analysis of effects of a healthcare intervention. In this study, ‘costing study’ refers to the cost analysis within an economic evaluation. Costing studies can be performed across a multitude of delivery settings, such as hospitals or primary care centers, and at different levels ranging, for example, from a whole hospital (6–9) or hospital unit (10, 11) to a specific service within a hospital (11–14). The type of costing study chosen is dependent on the research question of the study, also known as the ‘decision problem’. Examples of decision problems could be profitability of a hospital or resources utilization within a specific department. Disease-specific costing provides healthcare providers, policy makers, and insurance companies with valuable information on a variety of decision problems in healthcare, such as the assessment of resource requirements to provide a specific service, the assessment of financing and delivery inefficiencies, or the identification of cost areas for cost reductions (1, 3). To facilitate the implementation of costing studies in healthcare, many high-income countries have established health economic guidelines (3, 6–8). However, the overriding constraint with conducting disease-specific costing studies in low- and middle-income countries (LMICs) is data availability (1, 9–14), including the lack of accurate financial records and incomplete patient disease registers, and a lack of expertise to conduct costing studies. There are very few manuals for costing studies in LMICs, and those published do not focus primarily on disease-specific costing (4, 6).

Health economic guidelines from high-income countries usually comprise a stepwise process to support decisions regarding the methods of data collection and analysis. The steps often followed include choice of the perspective of the cost study, choice of cost categories (unit of analysis), identification of cost items, measuring cost items, valuing cost items, and dealing with uncertainty (3, 6–8, 15–17) . Each of these steps entails a trade-off between accurate, patient-level cost estimates, and data availability constraints. Health economic guidelines of high-income countries (3, 7, 8) make use of high quality, freely available data, which is often unavailable in LMICs resulting in the unsuitability of such guidelines. Therefore, this paper describes a step-by-step guideline to conducting disease-specific costing studies in settings with limited data availability. The step-by-step guideline is based on existing guidelines from high-income countries (3, 7, 8) and the World Health Organization (6) but adjusted, based on observation in the field, to a user-friendly guideline that is feasible to use in settings with limited data availability and resources. A secondary objective is to illustrate how each of these steps was applied in a study of cardiovascular disease (CVD) prevention care in rural Nigeria.

## Step-by-step guideline for disease-specific costing

This step-by-step guideline provides practical recommendations on methods and data sources in settings with data availability constraints for six sequential steps: 1) definition of the study perspective, 2) characterization of the unit of analysis, 3) identification of cost items, 4) measurement of cost items, 5) valuation of cost items, and 6) uncertainty analyses. This section describes each of these steps, and the next section provides more details about steps (4) measurement of cost items and (5) valuation of cost items.

A description of each step is immediately followed by an illustrative example, applied to Ogo Oluwa Hospital (OOH) in rural Nigeria. OOH is a private primary healthcare center in Bacita, a rural community in Kwara State, Nigeria. OOH has admission capability and operates primary care outpatient clinics. In addition, the QUality Improvement Cardiovascular Care Kwara-I (QUICK-I) project was rolled out in OOH in 2010. A description of the QUICK-I project can be found in detail elsewhere (18). In short, the project aims to evaluate the operational and financial feasibility of providing CVD prevention care according to international guidelines. The project follows a cohort of 349 patients who are treated for CVD risk factors, i.e. hypertension or non-insulin-dependent diabetes. Our illustrative example applies to the hospital's CVD prevention outpatient clinic, where this step-by-step guideline is currently applied to the costing component of the project.

### Step 1: The perspective of the cost study

The decision problem determines the perspective of the cost study. For example, if a policy maker wants to calculate the cost-effectiveness of treating a specific illness, productivity losses due to illness may need to be taken into consideration (15, 17). This societal perspective in which costs outside the formal healthcare system are also considered is often not feasible in LMICs due to data availability constraints. A provider perspective is used when the cost per patient incurred by the healthcare provider (such as a hospital) is under analysis. Up until now, cost studies in LMIC usually rely on the provider perspective, third party payer perspective (e.g. state-sponsored, or insurance) or patient perspective (1, 2, 4, 19–21).

#### Illustrative example

The decision problem of the QUICK-I study relates to the resource requirements of providing CVD prevention care in the hospital. This decision problem led to the choice of a provider perspective in which only the costs incurred by OOH were considered. Items such as travel costs of patients, lost labor market productivity, and other societal costs were not taken into account.

### Step 2: Unit of analysis

The second step is the determination of the unit of analysis, which is also reliant on the decision problem. If the decision problem is the profitability of a hospital or department, the single hospital (9–12), or single hospital department (14, 22), would be the most appropriate unit of analysis, respectively. If the decision problem relates to the payout by an insurance provider for treatment of a specific disease, the unit of analysis should be disease-specific (13, 23). As an increasing number of LMICs are gradually implementing Diagnosis Related Group (DRG) systems, DRGs may be a particularly appropriate unit of analysis for disease-specific costing (17). DRGs are clinically meaningful groups of patients that have the same diagnosis and similar patterns of resource consumption (24). Alternatively, clinical pathways based on guidelines or daily clinical practice in a hospital can be used to support disease-specific costing. Within each unit of analysis specific healthcare service activities can be identified, such as consultations and diagnostic tests.

#### Illustrative example

**Fig. 1:**
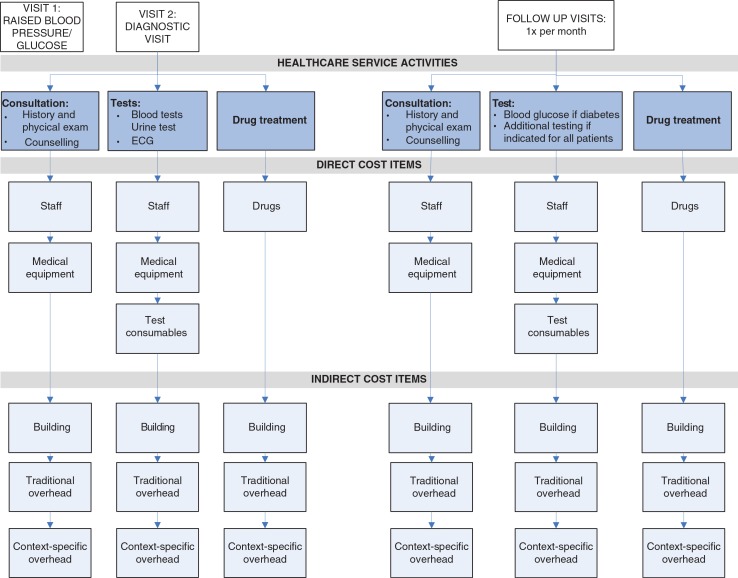
Clinical pathways with relevant healthcare service activities and cost items for CVD prevention care. Note: Each visit consists of different healthcare service activities (consultations, diagnostic tests, drugs). Each healthcare service activity consists of direct cost items and indirect cost items as listed in the figure. Traditional overheads include: security and housekeeping staff, information technology, building and equipment maintenance, fuel and electricity cost, training, medical non-CVD specific equipment, and general equipment. Context-specific overheads include: pharmacy staff, administration staff, general consumables (non-test specific).

As DRGs are not available in Kwara State, clinical pathways for CVD prevention care were developed based on international guidelines for CVD prevention care. Figure 1 shows this clinical pathway with the identified healthcare service activities for CVD prevention care, i.e. consultations, tests, and drug treatment.

### Step 3: Identification of cost items

**Fig. 2:**
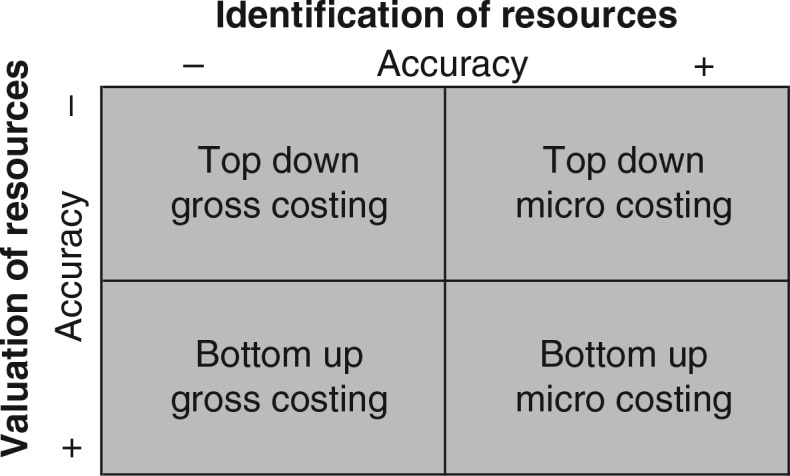
Methodology matrix and the level of accuracy at the identification and valuation of cost components.

In Step 2, healthcare service activities were identified (such a consultations and diagnostic tests). Each healthcare service activity comprises different cost items such as staff, equipment, consumables, and drugs. Depending on the level of detail desired or available, the number of cost items contained within a healthcare service activity will vary. ‘Gross-costing’ requires relatively few resources but provides a limited level of detail (3, 20, 25) compared to ‘micro-costing’, which provides a high level of detail but is resource and data intense (see Fig. 2). An example of gross-costing is if 20% of the hospitals patient population receive treatment for CVD prevention care, 20% of total hospital costs will be assigned to CVD prevention care. On the other hand, micro-costing would identify cost items in as much detail as possible.

#### Illustrative example

Micro-costing was employed to arrive at the most precise cost estimates. Each healthcare service activity was divided into direct costs and indirect costs. Direct cost items include staff involved in direct patient care, equipment that could be assigned to a specific treatment, consumables for specific tests, and drugs, while indirect cost items include building and overheads (Fig. 1). As not all cost items could be determined at the level of detail desired, a context-specific overhead category was created. This included, for example, pharmacy staff whose time would usually be one of the direct patient care staff cost items. However, since their time could not be specifically assigned to CVD prevention care or any other disease-specific activities, these costs were included as part of the context-specific overhead category.

### Step 4: Measurement of cost items

Measurement of cost items refers to measuring volumes of patient utilization for the different cost items. For example, a consultation may take 15 min of a doctors’ time, 10 min of a nurse's time, make use of certain equipment, and result in a number of drugs being dispensed. Under gross-costing this step can be ignored, as one does not measure individual cost items. The availability and level of precision of patient utilization data usually differs between cost items. Data often needs to be collected from multiple data sources (e.g. patient registers and records, expert opinion, laboratory and pharmacy registers, insurance utilization databases, databases of ongoing studies). Where patient utilization data is not available, data collection tools need to be developed (e.g. Staff Activity Log forms, in which staff reports time spent per patient). There can also be other expenditures (e.g. capital projects) that must be accounted for in the data collection process and in the data analysis. The next section expands on the measurement methods available for each individual cost item as well as provides examples within the illustrative example.

### Step 5: Valuation of cost items

Cost items can be valued using two approaches: top-down or bottom-up (Fig. 2). The top-down approach relies on comprehensive sources, such as annual financial accounts, and divides aggregated costs by the total number of patients. For example, annual hospital expenditures on CVD prevention care drugs divided by the number of CVD prevention care patients leads to a cost estimate of drugs for the average patient (3, 25, 26). To value cost items using the bottom-up approach, patient utilization data needs to be multiplied by unit prices, leading to cost estimates for individual patients (3, 25, 26). Possible data sources to obtain unit prices include hospital inventories, suppliers, and manufacturers or international sources. The next section will expand on the bottom-up valuation methods available for each individual cost item.

### Step 6: Dealing with uncertainty

All cost estimates are subject to uncertainty (3, 27). An important level of uncertainty concerns parameter uncertainty. This arises due to variation around estimates of variables, such as staff time spent per patient, and due to assumptions on, for example, missing data. The impact of parameter uncertainty should always be assessed using deterministic univariate or multivariate sensitivity analyses (3, 27–29). In univariate sensitivity analyses, one parameter is changed at a time while holding the other constant. Cost estimate parameters can be varied with a fixed percentage or uncertainty ranges such as standard deviations. Alternatively, point estimates can be varied based on parameter distributions. Multivariate sensitivity analyses vary more parameters at the same time.

Other important levels of uncertainty include model uncertainty and generalizability uncertainty (6). Dealing with these uncertainties falls outside the scope of this paper.

#### Illustrative example

Table 1 presents examples of univariate sensitivity analyses on a selection of parameters with a high level of uncertainty. In this example, a fixed percentage of 20% was used in all cases, but the choice in parameter variation is often an arbitrary choice in the absence of good quality information. Depending on the level of accuracy of the data, other percentages could be used and the percentage can be varied per parameter.

**Table 1:** Illustrative example of parameter uncertainties, alternative assumptions, and sensitivity analyses

Assumption	Alternative assumption	Sensitivity analysis
Mark-up % similar for all services assuming linear relation between direct and indirect costs	CVD prevention patients might ‘consume’ less building and overhead costs because they are seen in an outpatient setting	Decrease building mark-up% and traditional overhead mark-up% by 20%
Pharmacy staff and administrative staff in indirect costs, assumes that time spent per patient is similar for all patients	CVD prevention patients might use more pharmacy resources: almost all patients on drugs and often complicated drug regimes. Similar consideration for record staff (searching for files of chronic patients)	Increase ‘context-specific’ overhead including records and pharmacy mark-up% by 20%
CVD consultation: doctor costs based on salary average medical staff	In this setting there is a lack of doctors. In private clinics, the medical director tends to run also patient clinics. In OOH, usually three doctors including the medical director attend to all patients	Use both medical director salary (1/3) and regular doctor salary (2/3) to value costs per minute for a doctor
Productive working hours based on contract hours	Certain staff such as doctors are overburdened and work many more hours per week	Increase productive work hours by 20%
Interest rate=16% (Central Bank)	Local interest rate in rural setting for entrepreneurs might be much higher (personal communication: can be up to 25%)	Vary by 20%
Building value: estimated to be 8% of building maintenance	No other data available	Vary building value mark-up by ±20%

CVD: cardiovascular disease; OOH: Ogo Oluwa Hospital.

## Measurement and valuation of cost items

The combination of the bottom-up approach and micro-costing (bottom-up micro-costing) is considered to result in the most accurate cost estimates for healthcare service activities because all cost items are identified and valued at the most detailed level. However, bottom-up micro-costing is time and resource consuming and may not always be feasible (1, 3, 26). Therefore, a mixed methodology of costing is recommended (4). The mixed methodology of costing applies bottom-up micro-costing to activities which account for a large share of total costs or to activities for which data collection is reasonably feasible. Less accurate methods can be used for the remaining activities (i.e. top-down micro-costing and/or gross-costing; Fig. 2). In this way, the costing methodology may be tailored to the decision problem in question. Table 2 presents the methods, data requirements, and calculations to measure and value cost items for disease-specific costing using a mixed methodology.

**Table 2:** Methodologies, data requirements, and calculations to measure and value cost items for disease-specific costing

Cost component	Methodology	Data requirements	Calculations
Direct costs			
Staff	Bottom-up micro-costing	Staff salary for staff involved in care for specific disease	1) Yearly staff salary/yearly productive work minutes=staff costs per minute
		Productive working hours staff	2) Minutes spent per patient with specific disease×
		Minutes spent per patient with specific disease	staff costs per minute
	Top-down micro-costing	Staff costs for specific disease	Total staff costs for specific disease/total number of
		Total number of activities (e.g. consultation for CVD prevention care)	patients with specific disease
Equipment	Bottom-up micro-costing	Equipment prices (purchasing value)	1) PMT^a^=(equipment prices, life years, interest rates)
		Interest rate Life years Maintenance costs Total yearly equipment utilization all patients Utilization per patient with specific disease	2) (Annuity+ maintenance costs)/total yearly equipment utilization=equipment cost per test 3) Equipment cost per test×utilization per patient with specific disease
	Top-down micro-costing	Equipment prices Interest rate Life years Maintenance costs Total number of patients	1) PMT^a^ =(equipment prices, life years, interest rates) 2) (Annuity + maintenance cost)/total number of patients
Drugs and Consumables	Bottom-up micro-costing	Unit prices per drug/consumableUtilization per patient with specific disease	Unit prices per drug and consumable×utilization per patient with specific disease
	Top-down micro-costing	Total drug and consumable costs for specific disease	Total drug and consumable costs for specific disease/total number of patients with specific disease
		Total number of patients with specific disease	
Indirect costs			
Building and overhead	Mark-up	Total direct costs hospital Total indirect costs hospital	Total direct costs hospital/total indirect costs hospital
	Inpatient day	Total indirect costs hospital Total number of inpatient days	Total indirect costs hospital/total number of inpatient days
			

a PMT is an annuity calculation function in Microsoft Excel.

### Staff

As discussed above, bottom-up micro-costing requires patient utilization data to be multiplied by unit prices, leading to cost estimates for individual patients. To calculate staff costs using this approach, staff salaries, productive working hours, and minutes spent per patient are required. When calculating the staff cost per consultation, for instance, the number of minutes spent per patient should be combined with staff costs per minute. While salaries are usually available, the standard productive working hours used in high-income countries (30, 31) often do not apply to LMICs and minutes spent per patient are usually not available. Real productive working hours and minutes spent per patient are preferably observed by means of time and motion studies (staff and patient observations), which can ascertain staff time with the most accuracy, especially with regards to determining staff idle time and time spent on healthcare service activities other than direct patient care (20, 23). Alternatively, more practical techniques such as Staff Activity Log forms (self-reported time registration) or staff interviews may be used (20).

#### Illustrative example

The bottom-up approach was used to allocate the salary of doctors, nurses, and laboratory staff to healthcare service activities. Unit costs per hour were calculated by dividing their yearly salary by the number of productive working hours per year. Productive working hours were based on average number of shifts per month and shift length, taking into account official leave days and an estimation of the annual number of sick days for nurses, doctors, and laboratory staff. A simple data collection tool was developed to track staff activity (Table 3). This tool included a Staff Activity Log form in which staff reported all of their activities, time required to perform these activities and the number of patients attended. Self-reported data were verified through interviews and direct observations by researchers and adjusted if needed. Costs for pharmacy and administrative staff were included in the overheads, since it was not feasible to record staff activities that specifically related to CVD prevention care.

**Table 3:** Examples of data collection tools

Data tool	Data collected
Staff activity	
General information	Staff function, start time shift, end time shift
Regular daily activities:	For each activity:
Ward round, consultation, surgery, administrative tasks	Start time/end time, number of patients, patient category (e.g. disease based)
Extra activities:	For each activity:
Emergency consultations, emergency surgery, deliveries, ECG recording, ultrasound, other specify	Start time/end time, number of patients, patient category (e.g. disease based)
Monthly hospital expenditures	
Staff	Staff salary per function
Drugs	Expenditures per drug category
Consumables	Expenditures for laboratory consumables, other consumables
Building maintenance	Monthly building maintenance expenditures
Equipment	Purchase and maintenance expenditures
Other overhead	Training, information technology, communication expenditures, stationery, fuel, and electricity

### Equipment

Equipment costs per test are preferably calculated based on annualized equipment replacement value (determined through an annuity function) and yearly maintenance costs divided by the number of tests performed per year (4). The annuity function to determine the annualized equipment replacement value takes into account the purchasing price of the equipment, the life expectancy (in life years), and the interest rate. Replacement values (present purchasing value) can be obtained from hospital accounts, local suppliers, or manufacturers. Alternatively, historical values can be used. Country-specific interest rates can be obtained from (inter)national sources such as the World Bank (32), the International Monetary Fund (33) or national central banks. Interest rates may also be obtained from local banks. However, these interest rates might be highly volatile in LMICs and therefore very context-specific. The WHO-CHOICE project provides standard life-years for general equipment such as computers and generators (34), but manufacturers can also provide information on life years of specific medical equipment. Yearly maintenance costs and the number of tests performed per year may be acquired from hospital financial statements and patient and laboratory registers.

If the number of tests performed per year is not available, annuity and maintenance costs may be divided by the total number of patients, but this assumes that all patients use the equipment equally. Alternatively, equipment costs could be regarded as part of the overhead costs.

#### Illustrative example

Cost of medical CVD prevention-related equipment included costs of blood pressure devices, scales and stadiometers, stethoscopes, ophthalmoscopes, two laboratory devices for blood and urine tests, and an ECG machine. International sources, such as the WHO-CHOICE data (6, 34) and manufacturers, were used to get the equipment value and useful life-years and the Central Bank of Nigeria interest rate was uniformly applied as the interest rate (35).

The number of tests performed per year was available for the patients included in the QUICK-I cohort but not available for other patients in OOH making use of this equipment, which had to be estimated based on staff interviews and the number of other patients. Utilization data for medical non-CVD specific equipment such as examination tables as well as general equipment such as computers and generators was not available. Therefore, the annuity of this equipment was included in the overhead costs.

### Drugs and consumables

Bottom-up micro-costing requires unit prices of drugs, consumables, and patient utilization data. Unit prices are usually available from hospital suppliers but individual patient utilization data may be more difficult to obtain. If no database is available, patient records and pharmacy and laboratory registers may provide valuable information but if these are not electronic, data collection can be very time consuming.

#### Illustrative example

Where the hospital supplier provided itemized prices for drugs and consumables, the QUICK-I study provided data on patient drug utilization and test-specific consumable utilization.

However, because itemized prices and individual patient utilization were not available for general consumables such as syringes and cotton swabs, yearly general consumable costs determined from hospital administration records were included in the overhead costs.

### Building

Similar to the calculation of equipment costs, building costs are preferably determined by means of annuity costs and maintenance costs. Annuity costs include the present value of the building (3, 4, 36). As the present value of the building is often not available in LMICs, the building value may be estimated based on the yearly building maintenance costs. For example, building maintenance can be assumed to be 8% of the building value (36). Yearly maintenance costs may be acquired from annual financial accounts. Another method to determine the present value of the building is to derive this from the rental value of the building.

Two methods can then be used to include the yearly building costs in the estimated costs per service: assigning costs directly to the service or including the building costs in the overheads. If the building costs are to be assigned to a healthcare service activity, such as a consultation, the number of square meters of the hospital used for that activity, as opposed to other activities provided by the hospital, needs to be determined. Thereafter, the sum of annuity costs and yearly maintenance costs of this section of the hospital should be divided by the number of consultations per year (36). The number of activities performed per year (i.e. consultations) can be determined from patient utilization databases. Alternatively, the annual building and maintenance costs can be assigned to the general overheads. Overheads are discussed in more detail in the next section.

#### Illustrative example

The value of the building or rental prices was not available. Therefore, we assumed that the yearly building maintenance costs were 8% of the building value. The number of useful life-years of the building was assumed to be 25 years; the Central Bank of Nigeria interest rate of 16% was applied (mean interest rate for the study period) (35). As resource utilization of the hospital building could not be assigned to each or any healthcare service activity, the annuity of the building and yearly maintenance costs were included in the overheads.

### Overhead

There are several methods to allocate overhead costs to disease-specific direct costs. A mark-up method involves calculating a mark-up percentage by dividing the yearly total hospital direct costs by the yearly hospital indirect costs and then increasing the disease-specific direct costs by this mark-up percentage. The mark-up percentage allocation method assumes a linear relationship between direct and indirect costs, making it a simple and feasible method for LMICs (3, 4, 31). However, this assumption has its limitations. For example, if a specific service requires more than the average amount of overhead costs, the actual indirect costs will be underestimated. Another method often used for the allocation of overhead costs is inpatient day allocation, in which all patients are assumed to have the same indirect costs per inpatient day, regardless their actual resource use. Although alternative methods exist, such as weighted service and hourly rate allocation (31), these are very time consuming and therefore not feasible for LMICs.

#### Illustrative example

Overheads were reported separately for traditional and context-specific overheads. Traditional overheads included security and housekeeping staff, information technology, building and equipment maintenance, fuel and electricity cost, training, medical non-CVD specific equipment and general equipment. Context-specific overheads included staff involved in direct patient care such as the pharmacy staff and administrative staff but for whom it was not feasible to measure resource use for CVD prevention care specifically. The same applied to general consumables such as cotton swabs and syringes. Overheads were allocated to the direct costs using a mark-up percentage. The mark-up method was chosen because CVD prevention care is provided on an outpatient basis, which makes inpatient day allocation less suitable. Total direct and indirect hospital costs were obtained from the monthly hospital expenditures sheet (Table 3). Had, for example, the total yearly direct costs of the hospital amounted to US$ 100,000, and the total yearly indirect costs of the hospital amounted to US$ 50,000 (composed of building costs US$ 9,000, traditional overheads US$ 27,000 and context-specific overheads US$ 14,000), the mark-up percentage would be 50%. All disease-specific direct costs would have to be increased by 50% to include overhead costs.

## Discussion

We present a step-by-step guideline, which is a feasible, user-friendly tool for disease-specific costing in LMICs. The step-by-step guideline is based on health economic costing guidelines but demonstrates the need and ability to adapt to settings, for example, with limited data availability. These guidelines will vary depending on the country-specific context. The step-by-step guideline presented in this paper can serve as a manual for hospital managers and policy makers in healthcare. Cost analyses provide a critical input into economic evaluations, which is crucial to evaluate and understand the overall value of treatment programs, interventions, and healthcare policies.

The main limitation in conducting costing studies in LMICs concerns limited and poor quality patient utilization data, which are vital requirements for measuring the identified cost items (9–14). The aim would be to minimize data collection while maximizing the ability to measure the difference in costs between patients (37). The most feasible approach to achieving this aim is by making use of a mixing-methods costing approach. Consistent with previous studies (27, 31), we recommend applying micro-costing to those cost items that comprise a large share of total costs. When local data is inconsistent or inadequate, there are possibilities to create data collection templates, conduct interviews, and tailor existing hospital data sources to conduct patient-level analysis (Table 3). For example, in the case of the QUICK-I project, there was no hospital financial registration system and annual financial accounts were lacking. A simple sheet in Excel was developed to track monthly hospital expenditures to overcome this issue.

The clinical pathway approach presented in the QUICK-I illustrative example presents a clear outline of the hospital services (i.e. consultation, drug treatment, laboratory testing) where costs are incurred. This is a novel approach for disease-specific costing that allows comparison with cost estimates in other settings that may be following the same guidelines. The pathway approach also allows calculating costs for different scenarios. For instance, under the QUICK-I project, diagnostic tests are done frequently, up to the standards of high-income countries. However, WHO guidelines allow for less frequent testing in lower resource settings, so costs can be recalculated by adjusting the frequency of tests. Furthermore, clinical pathways provide a valuable comparator to actual practice in those settings where guidelines are not being followed.

## Conclusion

A mixed costing methodology can be a viable alternative to standard, top-down, and gross-costing approaches that are often used in LMICs settings due to (the assumed) lack of data availability. The flexibility of a mixed methodology allows using all available data sources and allows making tradeoffs between more accurate bottom-up micro-costing and more feasible approaches to measure cost items. This leads to comprehensive disease-specific patient-level cost estimates. This paper can be used as a guide for hospital managers and healthcare planners in LMICs to conduct feasible, disease-specific cost analyses with the most accuracy possible given a lack of comprehensive data sources.
